# Evaluation of the Autof ms1000 mass spectrometry for rapid clinical identification of filamentous fungi

**DOI:** 10.1186/s12866-023-02968-w

**Published:** 2023-08-22

**Authors:** Keping Ao, Xiaohan Li, Weili Zhang, Zhixing Chen, Ya Liu, Ling Shu, Yuling Xiao, Siying Wu, Yi Xie, Mei Kang

**Affiliations:** Department of Laboratory Medicine, West China Hospital, Sichuan University, No. 37 Guoxue Lane, Chengdu, 610041 China

**Keywords:** MALDI-TOF, Autof ms1000, Mass spectrometry, Filamentous fungi, Isolates identification, Accuracy

## Abstract

**Background:**

Matrix-assisted laser desorption/ionization time-of-flight mass spectrometry (MALDI-TOF MS) has revolutionized microbial identification. However, there is a lack of data on its performance in identifying filamentous fungi. The objective of our study was to evaluate the accuracy of the Autof ms1000 mass spectrometry for identifying filamentous fungi in the clinical microbiology laboratory.

**Results:**

Among 106 samples tested using the Autof ms1000 system, 101 (95.28%) were identified at the genus or species level, and 81 (76.41%) were accurately identified at the species level. Additionally, we developed a new rapid formic acid extraction method with simple pretreatment for filamentous fungi that saved time and provided accurate results.

**Conclusions:**

The Autof ms1000 mass spectrometer proved to be a valuable tool for identifying filamentous fungi. However, upgrading the database is recommended for correctly identifying rare strains.

**Supplementary Information:**

The online version contains supplementary material available at 10.1186/s12866-023-02968-w.

## Background

Accurate microbiological identification plays a crucial role in diagnosing infectious diseases [1–3]. With the increasing number of immunosuppressed individuals, infections caused by filamentous fungi, such as invasive aspergillosis (IA), are becoming more prevalent and have high mortality rates [4]. Talaromycosis is another such infection that tends to occur in immunocompromised patients with acquired immunodeficiency syndrome (AIDS). Additionally, cases of invasive and disseminated fusarium infections have steadily increased over the past 20 years. However, identifying filamentous fungi is challenging as it requires professionals with significant experience in identifying fungi based on morphology.

In recent years, MALDI-TOF MS has been widely used for identifying conventional bacteria and common yeast with excellent sensitivity, high throughput, simple operation, low cost, quick results, and an extensive database, improving the turnaround time of microbiology reports [5]. Accurate identification of filamentous fungi using mass spectrum analysis is crucial. Some studies have used the Bruker Microflex or the Biomerieux Vitek MS for identifying filamentous fungi. However, limitations in the library or imperfect spectra resulted in more than 20% of molds remaining unidentified at the species level using Bruker Microflex libraries [6]. Additionally, Biomerieux Vitek MS was unable to identify 10.5% of the samples [7]. Given the slow growth and conventional identification time of filamentous fungi, the severity of most fungal infections, and the complexity of some pretreatment methods for mass spectrum identification, a simple pretreatment method is urgently needed.

There are currently some non-culture-based methods used in clinical settings for identifying filamentous fungi, such as nucleic acid-based or serology-based methods, and the detection of extracellular glycoproteins, galactomannan, and Beta-1,3-glucanases. However most of these methods are nonspecific or can only identify fungi upto genus level. Therefore, the development of more specific detection methods is crucial. Fluorescent in situ hybridization-based molecular methods may be a suitable alternative to achieve this purpose [8]. However, the lack of standardization limits its utility [9].

Since 2018, the Autof ms1000 has been successfully used in microbiological laboratories for routine bacteria identification [10, 11]. However, its use for identifying filamentous fungi has been limited. Therefore, the aim of the present study was to evaluate the ability of the Autof ms1000 to rapidly identify filamentous fungi in a clinical microbiology laboratory setting. Additionally, a new and simple pretreatment method was explored in this study.

## Results

The results of identification using the routine pretreatment method were as follows: the same 5 strains(*Beauveria bassiana, Cunninghamella, Fonsecaea pedrosoi, Geomyces sp*, and *Phoma sp)*were not reliably identified, while 83.02% (88/106) were correctly identified at the species level, and 95.28% (101/106) were correctly identified at the genus level(Table 1). Specific results can be found at Table S1.

**Table 1:** Result analysis with filamentous fung

		*Autof* ms1000 Correct identification to the level			
		Simple pretreatment method		Routine pretreatment method	
Genus	Species(n = 106)	Species	Genus	Species	Genus
***Aspergillus***	*Aspergillus fumigatus*(n = 23)	23		23	
	*Aspergillus flavus*(n = 10)	9	1	9	1
	*Aspergillus clavatus*(n = 2)		2	1	1
	*Aspergillus nidulans*(n = 1)	1		1	
	*Aspergillus terreus*(n = 9)	8	1	8	1
	*Aspergillus ustus*(n = 2)	2		2	
***Fusarium***	*Fusarium oxysporum*(n = 1)	1		1	
	*Fusarium solani*(n = 3)	2	1	2	1
	*Fusarium proliferatum*(n = 2)	1	1	2	
***Trichophyton sp***	*Trichophyton tonsurans*(n = 6)	5	1	6	
	*Trichophyton rubrum*(n = 1)		1	1	
	*Microsporum gypseum*(n = 2)	1	1	1	1
***Mucor***	*Mucor hiemalis*(n = 1)	1		1	
	*Mucor ramosissimus*(n = 1)		1		1
	*Lichtheimia ramosa*(n = 1)	1		1	
	*Rhizopus formosensis*(n = 1)		1		1
***Other***	*Talaromyces marneffei*(n = 12)	10	2	11	1
	*Chaetomium globosum*(n = 2)	2		2	
	*Sporothrix schenckii*(n = 7)	4	3	5	2
	*Exophiala dermatitidis*(n = 4)	1	3	1	3
	*Sarocladium strictum*(n = 2)	1	1	2	
	*Scopulariopsis brevicaulis*(n = 1)	1		1	
	*Scedosporium prolificans*(n = 1)	1		1	
	*Alternaria alternata*(n = 3)	3		3	
	*Paecilomyces variotii*(n = 1)	1		1	
	*Penicillin citrinum*(n = 1)	1		1	
	*Trichothecium roseum*(n = 1)	1		1	
	*Beauveria bassiana*(n = 1)		N		N
	*Cunninghamella*(n = 1)		N		N
	*Fonsecaea pedrosoi*(n = 1)		N		N
	*Geomyces sp*(n = 1)		N		N
	*Phoma sp*(n = 1)		N		N
**All**	106	81	20	88	13

The results obtained using the new and simple pretreatment method are as follows: out of 106 strains of filamentous fungi, 101 (95.28%) were correctly identified at the genus level, and 81 (76.41%) were correctly identified at the species level. However, there were 5 strains, namely *Beauveria bassiana, Cunninghamella, Fonsecaea pedrosoi, Geomyces sp*, and *Phoma sp*, that could not be reliably identified(Table 1). Specific results can be found at Table S2.

## Discussion

Pinheiro et al. evaluated the ability of VITEK MS (a MALDI-TOF MS system) to identify filamentous fungi and differentiate species within a complex, resulting in 47 out of 74 (63.5%) correct identifications at the species level [12]. Although it may seem that Autof is superior to VITEK MS, in another study, Sun et al. [13] evaluated three different MALDI-TOF MS systems for identifying clinically relevant filamentous fungi. They found that VITEK MS identified 96.0% at the species level, Biotyper identified 42.1% at the species level, and Autof identified 58.7% at the species level. This suggests that Autof was not superior to VITEK MS, but it was superior to Biotyper. This difference in performance could be related to the different strains used in each study.

The present study covered a broad range of filamentous fungi, including *Aspergillus spp, Mucor spp, Fusarium spp, Trichophyton spp, Chaetomium globosum, Sporothrix schenckii, Alternaria alternata, Exophiala dermatitidis, Sarocladium strictum, Talaromyces marneffei*. *Chaetomium globosum, Microsporum, gypseum, Scopulariopsis, brevicaulis, Scedosporiumprolificans, Alternaria alternata, Paecilomyces variotii, Penicillin citrinum*, and *Trichothecium roseum* were identified at the species level, while *Mucor spp, Sporothrix schenckii, Exophiala dermatitidis, Talaromyces marneffei* were identified at the genus level.

Our results showed that the Autof system performed well in identifying Aspergillus spp, with most of them being identified at the species level. Patients with Aspergillus keratitis have a higher risk of perforation and are more likely to require surgery than those with Fusarium keratitis [14, 15]. In this study, we found no misjudgment between the identification of Aspergillus and Fusarium, and the accuracy of both genera was higher than that of other systems. The Bruker Biotyper MALDI-TOF MS system was able to identify only 30.2% of Aspergillus isolates to the species level and 49.3% to the genus level [16]. Moreover, the Bruker Biotyper misjudged the identification of Fusarium [17]. However, the data size of our study was limited, which implies some restrictions.

The Autof MS system is very useful for identifying the most common clinical isolates of filamentous fungi. After the target board is placed in the instrument, the time from the beginning of data collection to the identification result is short, taking only about 40 s to vacuum, less than 3 s to collect, and about 5 s to produce an identical result. In contrast, Bruker requires 5 min to vacuum, more than 3 s to collect, and more than 5 min to produce an identical result. Additionally, a single piece of software can perform multiple operations for Autof, while Bruker requires three interfaces, and manual mode is even more complicated.

The differences in results of different identification systems may also be due to the pretreatment method used, where in-tube lysis is generally more efficient than on-plate lysis. However, the in-tube lysis method used by Biotyper and VITEK can be time-consuming and complex. In contrast, the new pretreatment method used by Autof MS was found to be easy to operate and time-effective, resulting in good accuracy.

The current gold standard for filamentous fungal identification is sequencing, which is not routinely performed in general laboratories. Conventional identification methods rely on morphological observation, which is time-consuming and requires experienced professionals. However, the new pretreatment method used by Autof MS was found to be accurate in identifying most clinical isolates of filamentous fungi at the species level (76.41%), with a correct species-level identification rate of 83.02% achieved by lowering the identification cutoff value.

In clinical laboratories, rapid identification methods are sometimes important, especially for critical patients. However, it is essential to ensure that the identification results are correct at least at the genus level, as infections caused by fungi of the same genus often require similar treatment. Rapid identification methods can be used for primary reporting, while traditional methods can be used for secondary reporting to achieve a balance.

The difficulty in identifying filamentous fungi lies in the challenge of protein extraction. Ya-Ting Ning et al. [18] developed two rapid protein extraction methods using focused-ultrasonication and zirconia-silica beads for filamentous fungi identification by MALDI-TOF MS, which significantly reduced sample processing time and demonstrated superior maximum and minimum S/N ratio, as well as comparable or superior identification to the routine method. However, more research is needed to compare different methods and systems. Overall, the new simple pretreatment method used by Autof MS could be a promising alternative to routine pretreatment methods in current use.

The accuracy of identification using mass spectrometry can be influenced by several factors, including the quality of protein extraction [19, 20] and database issues [21]. In this study, there were 5 strains that could not be reliably identified by Autof MS, namely *Beauveria bassiana, Cunninghamella, Fonsecaea pedrosoi, Geomyces sp*, and *Phoma sp*. Four of these strains were not present in the Autof database, while *Beauveria bassiana* was in the database, but the identified strain was different from the one in the database. Therefore, the current Autof database needs to be expanded to include a wider range of strains. Similar to the studies conducted by Honnavar et al. [22] and Paul [23], who expanded their database to identify Malassezia species and other fungi using MALDI-TOF MS, respectively, it is feasible to develop our own in-house database for Autof MS to improve its identification capabilities.

## Conclusions

Overall, the study demonstrated that the Autof ms1000 can be used as a rapid and reliable method for filamentous fungi identification in clinical routine microbiology laboratories. The new pretreatment method used in the study also showed promising results. However, it should be noted that the Autof ms1000 library may not cover all filamentous fungi, and further expansion and upgrading of the MALDI-TOF MS databases are necessary to ensure accurate identification of all isolates.

## Methods

### Microorganism isolates

We analyzed a total of 106 filamentous fungi strains, consisting of 74 clinical strains and 32 reference strains(Table S3). Clinical strains were isolated between 2019 and 2020 from the patients with fungal infections at the Department of Microbiology Laboratory Medicine, West China Hospital, Sichuan University. Reference strains were selected from the College of American Pathologists (2006–2015) and stored at − 80 °C. We inoculated one strain at three separate points on each Sabouraud dextrose agar (SDA) plate and incubated it to obtain three well-isolated colonies. We used SDA plates from AutoBio (Zhengzhou, China) in our laboratory. We cultivated Aspergillus spp, Penicillium spp, and Mucorales spp at 35 °C for 3 days and Fusarium spp and Sporothrix schenckii at 35 °C for 3–5 days, ensuring that the mycelia were clearly visible and could be harvested. The species used in the study are represented in the commercialized reference database.

### Molecular Identification of the clinical strains

The clinical strains, apart from the reference strains, were previously identified to the species level by using ITS [24–26] sequencing with the primers ITS1/ITS4(5’-TCCGTAGGTGAACCTGCGG-3’/5’-TCCTCCGCTTATTGATATGC-3’). Additionally, the b-tubulin and calmodulin sequencing techniques were also employed. For amplification of the b-tubulin gene, the Bt2a(5’-GGTAACCAAATCGGTGCTGCTTTC-3’) and Bt2b (5’-ACCCTCAGTGTAGTGACCCTTGGC-3’) primers were used, while the cmd5(5’-CCGAGTACAAGGAGGCCTTC-3’) and cmd6 (5’-CCGATAGAGGTCATAACGTGG-3’) primers were used for amplification of the calmodulin gene. The PCR amplification was performed using the TransGen AS111 PCR Kit (2 X EasyTaq SuperMix, CHINA). The PCR mix consisted of 2 X EasyTaq SuperMix 15 µL, 1 µL of each primer (forward primer, reverse primer), 1–2 µL of gDNA, and ultrapure water, with a total volume of 30 µL. The amplification was carried out in a thermal cycler (ABI, 2720, USA) with an initial denaturation step at 94 °C for 5 min, followed by 35 cycles of amplification (denaturation at 94 °C for 30 s, annealing at 55 °C for 30 s and extension at 72 °C for 1 min), and a final extension step at 72 °C for 5 min. In each reaction set, a non-template negative control was included using the Gel Electrophoresis apparatus (JUNYI, China). The sequencing was performed using the same primers used for amplification, and the obtained sequences were compared with NCBI databases.

## MALDI-TOF MS identification

The routine pretreatment method recommended by the manufacturer involved inactivating approximately one or two isolated colonies in 1 mL of 75% ethanol in a 1.5 ml Eppendorf tube. The mixture was vortexed and mixed, and then centrifuged at 13,000 g for 2 min. The ethanol was removed after another centrifugation at 13,000 g for 2 min. The supernatant was then disposed of, and the pellet was allowed to dry for 2–5 min at 37–40 °C. Protein extraction was accomplished by resuspending the pellet in 30–50 µL of formic acid. After allowing the mixture to sit for 5–10 min, 1 µL of the supernatant was spotted on the plate, dried at room temperature, and overlaid with 1 µL of CHCA matrix solution. The plate was dried at room temperature again and then placed into the instrument for testing.

A new and simplified pretreatment method was used in which one or two colonies were swabbed using a toothpick and mixed with 30 µL of lysate 1 (70% formic acid) in a 1.5 ml Eppendorf tube. The mixture was allowed to sit for 10 min, and 1 µL of the mixture was pipetted onto the plate and dried at room temperature. The isolates were handled in a biosafety cabinet until the matrix was dry, after which 1 µL of α-cyano-4-hydroxycinnamic acid (CHCA) matrix solution was overlaid on the sample. The CHCA matrix solution was prepared by dissolving one bottle of matrix solution dry powder (1.0 mg) in 60 µL of acetonitrile and 60 µL of 5% trifluoroacetic acid, following the instructions. The samples were dried again at room temperature and then placed into the instrument for testing.

The instrument was calibrated using a reference strain of Escherichia coli ATCC 25,922. According to the manufacturer’s recommendations for MALDI-TOF MS, the instrument needs to be quality controlled at least once a week. Samples used for quality control included positive quality control (E. coli ATCC 25,922, Pseudomonas aeruginosa ATCC 27,853, Staphylococcus aureus ATCC 29,213, Candida albicans ATCC 10,231) and negative quality control.

### Data analysis

The data obtained from Autof MS 1000 were analyzed using Autof Acquirer version 1.0.123 and Autof Analyzer version 1.0.50, library number v1.1.0, which includes 475 species (1800 MSPs) of filamentous fungi. The manufacturer’s interpretation criteria were followed, with a score of > 9.0 indicating species-level identification, a score between 6.0 and 9.0 indicating genus-level identification, and a score < 6.0 indicating unidentified fungi. Reproducibility tests were conducted for each fungus by performing three tests on a target plate to ensure consistency of the results. The results showed 100% consistency for 106 strains, at either the species or genus level. In cases where the results were not reliable, they were excluded from the analysis. It is important to note that this study only evaluated the commercially available database for clinical use and did not assess the in-house database or online databases for other research purposes.

### Electronic supplementary material

Below is the link to the electronic supplementary material.

**Table S1**? Autof ms1000 mass spectrometry identification results by Routine pretreatment.

**Table S2**? Autof ms1000 mass spectrometry identification results by Simple pretreatment

**Table S3**? the source and distribution of strains

## Data Availability

All data generated or analysed during this study are included in this published article and its supplementary information files.
