# Supplementary material for: Evaluation of the Autof ms1000 mass spectrometry for rapid clinical identification of filamentous fungi
Source: BMC Microbiol. 2023 Aug 22;23:228. doi: 10.1186/s12866-023-02968-w (PMC10464221; doi:10.1186/s12866-023-02968-w)
Supplement: Supplementary file 1 — Table S1? Autof ms1000 mass spectrometry identification results by Routine pretreatment. [file 12866_2023_2968_MOESM1_ESM.docx]

| Table S1：*Autof ms1000* mass spectrometry identification results by Routine pretreatment | |
| --- | --- |
| **Fungal name** | **Appraisal score** |
| *Aspergillus fumigatus* | 9.191 |
| *Aspergillus flavus* | 9.428 |
| *Aspergillus fumigatus* | 9.518 |
| *Mucor hiemalis* | 9.215 |
| *Aspergillus fumigatus* | 9.079 |
| *Aspergillus fumigatus* | 9.167 |
| *Aspergilus clavatus* | 9.406 |
| *Aspergillus fumigatus* | 9.456 |
| *Penicillin citrinum* | 9.53 |
| *Paecilomyces variotii* | 9.4 |
| *Exophiala dermatitidis* | 7.224 |
| *Exophiala dermatitidis* | 6.275 |
| *Sarocladium strictum* | 9.013 |
| *Sarocladium strictum* | 9.025 |
| *Chaetomium globosum* | 9.423 |
| *Talaromyces marneffei* | 9.324 |
| *Aspergillus nidulans* | 9.255 |
| *Sporothrix schenckii* | 9.159 |
| *Aspergillus flavus* | 9.621 |
| *Aspergillus fumigatus* | 9.458 |
| *Aspergillus fumigatus* | 9.417 |
| *Aspergillus terreus* | 9.191 |
| *Fusarium proliferatum* | 9.034 |
| *Fusarium proliferatum* | 9.292 |
| *Talaromyces marneffei* | 9.471 |
| *Exophiala dermatitidis* | 9.023 |
| *Talaromyces marneffei* | 9.534 |
| *Sporothrix schenckii* | 6.282 |
| *Trichothecium roseum* | 9.764 |
| *Fusarium oxysporum* | 9.451 |
| *Alternaria alternata* | 9.023 |
| *Trichophyton tonsurans* | 9.341 |
| *Aspergillus terreus* | 9.035 |
| *Alternaria alternata* | 9.132 |
| *Aspergilus clavatus* | 8.105 |
| *Aspergillus terreus* | 9.034 |
| *Aspergillus terreus* | 8.812 |
| *Aspergillus flavus* | 9.336 |
| *Aspergillus flavus* | 8.223 |
| *Aspergillus flavus* | 9.335 |
| *Aspergillus fumigatus* | 9.265 |
| *Aspergillus fumigatus* | 9.046 |
| *Aspergillus fumigatus* | 9.407 |
| *Exophiala dermatitidis* | 6.021 |
| *Talaromyces marneffei* | 9.476 |
| *Talaromyces marneffei* | 9.534 |
| *Aspergillus fumigatus* | 9.545 |
| *Trichophyton tonsurans* | 9.243 |
| *Trichophyton tonsurans* | 9.451 |
| *Lichtheimia ramosa* | 9.707 |
| *Sporothrix schenckii* | 9.359 |
| *Talaromyces marneffei* | 9.434 |
| *Trichophyton tonsurans* | 9.027 |
| *Beauveria bassiana* | N |
| *Aspergillus fumigatus* | 9.373 |
| *Aspergillus fumigatus* | 9.471 |
| *Aspergillus flavus* | 9.129 |
| *Aspergillus terreus* | 9.035 |
| *Aspergillus fumigatus* | 9.335 |
| *Aspergillus flavus* | 9.214 |
| *Cunninghamella* | N |
| *Aspergillus terreus* | 9.407 |
| *Aspergillus fumigatus* | 9.252 |
| *Aspergillus terreus* | 9.256 |
| *Aspergillus flavus* | 9.324 |
| *Aspergillus flavus* | 9.219 |
| *Fusarium solani* | 9.123 |
| *Aspergillus fumigatus* | 9.054 |
| *Aspergillus fumigatus* | 9.054 |
| *Aspergillus terreus* | 9.308 |
| *Aspergillus terreus* | 9.316 |
| *Aspergillus ustus* | 9.651 |
| *Aspergillus ustus* | 9.245 |
| *Fusarium solani* | 8.1 |
| *Trichophyton tonsurans* | 9.035 |
| *Trichophyton tonsurans* | 9.023 |
| *Aspergillus flavus* | 9.103 |
| *Aspergillus fumigatus* | 9.179 |
| *Aspergillus fumigatus* | 9.038 |
| *Fusarium solani* | 9.124 |
| *Microsporum gypseum* | 9.253 |
| *Aspergillus fumigatus* | 9.291 |
| *Trichophyton rubrum* | 9.013 |
| *Mucor ramosissimus* | 8.125 |
| *Rhizopus formosensis* | 6.264 |
| *Talaromyces marneffei* | 9.124 |
| *Talaromyces marneffei* | 8.5 |
| *Fonsecaea pedrosoi* | N |
| *Aspergillus fumigatus* | 9.139 |
| *Microsporum gypseum* | 8.449 |
| *Talaromyces marneffei* | 9.182 |
| *Talaromyces marneffei* | 9.121 |
| *Talaromyces marneffei* | 9.129 |
| *Chaetomium globosum* | 9.176 |
| *Aspergillus fumigatus* | 9.293 |
| *Talaromyces marneffei* | 9.057 |
| *Sporothrix schenckii* | 9.007 |
| *Sporothrix schenckii* | 9.159 |
| *Sporothrix schenckii* | 9.216 |
| *Sporothrix schenckii* | 8.252 |
| *Aspergillus fumigatus* | 9.387 |
| *Phoma sp* | N |
| *Geomyces sp* | N |
| *Scopulariopsis brevicaulis* | 9.319 |
| *Scedosporium prolificans* | 9.457 |
| *Alternaria alternata* | 9.234 |
