# Supplementary material for: Evaluation of the Autof ms1000 mass spectrometry for rapid clinical identification of filamentous fungi
Source: BMC Microbiol. 2023 Aug 22;23:228. doi: 10.1186/s12866-023-02968-w (PMC10464221; doi:10.1186/s12866-023-02968-w)
Supplement: Supplementary file 2 — Table S2? Autof ms1000 mass spectrometry identification results by Simple pretreatment [file 12866_2023_2968_MOESM2_ESM.docx]

| Table S2：*Autof ms1000* mass spectrometry identification results by Simple pretreatment | |
| --- | --- |
| **Fungal name** | **Appraisal score** |
| *Aspergillus fumigatus* | 9.271 |
| *Aspergillus flavus* | 9.048 |
| *Aspergillus fumigatus* | 9.256 |
| *Mucor hiemalis* | 9.018 |
| *Aspergillus fumigatus* | 9.422 |
| *Aspergillus fumigatus* | 9.42 |
| *Aspergilus clavatus* | 8.567 |
| *Aspergillus fumigatus* | 9.009 |
| *Penicillin citrinum* | 9.534 |
| *Paecilomyces variotii* | 9.197 |
| *Exophiala dermatitidis* | 7.144 |
| *Exophiala dermatitidis* | 6.237 |
| *Sarocladium strictum* | 8.017 |
| *Sarocladium strictum* | 9.037 |
| *Chaetomium globosum* | 9.3 |
| *Talaromyces marneffei* | 8.5 |
| *Aspergillus nidulans* | 9.52 |
| *Sporothrix schenckii* | 9.364 |
| *Aspergillus flavus* | 9.605 |
| *Aspergillus fumigatus* | 9.257 |
| *Aspergillus fumigatus* | 9.359 |
| *Aspergillus terreus* | 9.086 |
| *Fusarium proliferatum* | 8.95 |
| *Fusarium proliferatum* | 9.031 |
| *Talaromyces marneffei* | 9.086 |
| *Exophiala dermatitidis* | 9.03 |
| *Talaromyces marneffei* | 9.198 |
| *Sporothrix schenckii* | 6.104 |
| *Trichothecium roseum* | 9.707 |
| *Fusarium oxysporum* | 9.444 |
| *Alternaria alternata* | 9.016 |
| *Trichophyton tonsurans* | 9.252 |
| *Aspergillus terreus* | 9.133 |
| *Alternaria alternata* | 9.021 |
| *Aspergilus clavatus* | 7.325 |
| *Aspergillus terreus* | 9.08 |
| *Aspergillus terreus* | 8.535 |
| *Aspergillus flavus* | 9.04 |
| *Aspergillus flavus* | 7.4 |
| *Aspergillus flavus* | 9.293 |
| *Aspergillus fumigatus* | 9.265 |
| *Aspergillus fumigatus* | 9.355 |
| *Aspergillus fumigatus* | 9.316 |
| *Exophiala dermatitidis* | 6.037 |
| *Talaromyces marneffei* | 9.325 |
| *Talaromyces marneffei* | 9.142 |
| *Aspergillus fumigatus* | 9.086 |
| *Trichophyton tonsurans* | 9.121 |
| *Trichophyton tonsurans* | 9.105 |
| *Lichtheimia ramosa* | 9.114 |
| *Sporothrix schenckii* | 9.216 |
| *Talaromyces marneffei* | 9.309 |
| *Trichophyton tonsurans* | 9.026 |
| *Beauveria bassiana* | N |
| *Aspergillus fumigatus* | 9.162 |
| *Aspergillus fumigatus* | 9.123 |
| *Aspergillus flavus* | 9.265 |
| *Aspergillus terreus* | 9.061 |
| *Aspergillus fumigatus* | 9.303 |
| *Aspergillus flavus* | 9.231 |
| *Cunninghamella* | N |
| *Aspergillus terreus* | 9.086 |
| *Aspergillus fumigatus* | 9.035 |
| *Aspergillus terreus* | 9.079 |
| *Aspergillus flavus* | 9.406 |
| *Aspergillus flavus* | 9.29 |
| *Fusarium solani* | 9.102 |
| *Aspergillus fumigatus* | 9.191 |
| *Aspergillus fumigatus* | 9.151 |
| *Aspergillus terreus* | 9.167 |
| *Aspergillus terreus* | 9.034 |
| *Aspergillus ustus* | 9.045 |
| *Aspergillus ustus* | 9.238 |
| *Fusarium solani* | 7.99 |
| *Trichophyton tonsurans* | 9.012 |
| *Trichophyton tonsurans* | 8.236 |
| *Aspergillus flavus* | 9.049 |
| *Aspergillus fumigatus* | 9.458 |
| *Aspergillus fumigatus* | 9.265 |
| *Fusarium solani* | 9.036 |
| *Microsporum gypseum* | 9.079 |
| *Aspergillus fumigatus* | 9.471 |
| *Trichophyton rubrum* | 8.407 |
| *Mucor ramosissimus* | 7.941 |
| *Rhizopus formosensis* | 6.321 |
| *Talaromyces marneffei* | 9.027 |
| *Talaromyces marneffei* | 7.861 |
| *Fonsecaea pedrosoi* | N |
| *Aspergillus fumigatus* | 9.035 |
| *Microsporum gypseum* | 8.95 |
| *Talaromyces marneffei* | 9.028 |
| *Talaromyces marneffei* | 9.112 |
| *Talaromyces marneffei* | 9.114 |
| *Chaetomium globosum* | 9.026 |
| *Aspergillus fumigatus* | 9.061 |
| *Talaromyces marneffei* | 9.079 |
| *Sporothrix schenckii* | 9.012 |
| *Sporothrix schenckii* | 9.015 |
| *Sporothrix schenckii* | 8.883 |
| *Sporothrix schenckii* | 8.121 |
| *Aspergillus fumigatus* | 9.063 |
| *Phoma sp* | N |
| *Geomyces sp* | N |
| *Scopulariopsis brevicaulis* | 9.334 |
| *Scedosporium prolificans* | 9.451 |
| *Alternaria alternata* | 9.221 |
