# Supplementary material for: Evaluation of the Autof ms1000 mass spectrometry for rapid clinical identification of filamentous fungi
Source: BMC Microbiol. 2023 Aug 22;23:228. doi: 10.1186/s12866-023-02968-w (PMC10464221; doi:10.1186/s12866-023-02968-w)
Supplement: Supplementary file 3 — Table S3? the source and distribution of strains [file 12866_2023_2968_MOESM3_ESM.docx]

| Table S3： the source and distribution of strains | |
| --- | --- |
| reference strains （32）  from the College of American Pathologists (2006-2015) | *Aspergillus fumigatus（2007、2012）Aspergillus flavus（2008、2012）*  *Aspergilus clavatus（2013、2014）*  *Aspergillus nidulans（2012）*  *Aspergillus terreus（（2011）*  *Aspergillus ustus（2010）*  *Trichophyton rubrum（2007）*  *Chaetomium globosum（2014、2009）*  *Sporothrix schenckii（2007、2010、2015）*  *Exophiala dermatitidis（2007、2008）*  *Sarocladium strictum（2010）*  *Microsporum gypseum（2008）*  *Scopulariopsis brevicaulis（2010）*  *Scedosporium prolificans（2015）*  *Alternaria alternata（2006、2013、2014）*  *Paecilomyces variotii（2011）*  *Penicillin citrinum（2011）*  *Trichothecium roseum（2006）*  *Beauveria bassiana（2014）*  *Cunninghamella（2012）*  *Fonsecaea pedrosoi（2008）*  *Geomycessp（2008）*  *Phoma（2006）* |
| clinical isolates（74） | *Aspergillus fumigatus（21）*  *Aspergillus flavus（8）*  *Aspergillus terreus（8）*  *Aspergillus ustus（1）*  *Fusarium oxysporum（1）*  *Fusarium proliferatum（2）*  *Fusarium solani（3**）*  *Trichophyton tonsurans （6）*  *Microsporum gypseum（1）*  *Mucor hiemalis（1）*  *Mucor ramosissimus（1）*  *Lichtheimia ramosa （1）*  *Rhizopus formosensis（1）*  *Talaromyces marneffei（12）*  *Sporothrix schenckii（4）*  *Exophiala dermatitidis（2）*  *Sarocladium strictum（1）* |
